# Health Tract, No. 285

**Published:** 1867-03

**Authors:** 


HEALTH TRACT, No. 285.

### DRINK A DESPOTISM.

In February, 1866, a lady sent for a physician to see her husband, who was laboring under symptoms which she could not comprehend. He was a man with over three hundred thousand dollars, was highly esteemed, and had a most interesting family. To an ordinary eye, there was no special disease; there was no pain, there was no strength, no appetite, no enjoyment; but the physician, in the peculiar condition, discovered that the patient was laboring under the influence of long continued and incessant stimulation. He communicated his views to the wife as soon as an opportunity offered, and retired. The husband naturally desired to know the physician's opinion: "He said, my dear husband, that you were under the influence of constant stimulation, and that unless you renounce the habit, you connot live three months." "I can't do it," said he, and within the time he was buried. Every earthly consideration of family, fortune and friends appealed to him in vain, and failed to drag him away from his suicidal habits; to all their calling him from despotic indul gences, he could only respond:

"I can't do it!'

and yielding himself hopelessly to his captor, he perished in his prime.

It is related that a man, addicted to drink, was sent to the penitentiary for some crime; no liquor was allowed convicts, except by special medical direction, and every possible device having failed to secure him a supply, he came running to the keeper one day, holding out the bleeding stump of his arm, calling loudly for brandy to staunch the blood; in the flurry of the moment, a bowl was handed him, into which was thrust the gory stump, and the next instant he gulped the contents at a draught. Such are some of the despotisms of drink, and the only certain method of preventing one from falling under the influence of a tyrrany so terrible, is never to take a drop—such only are safe.

In 1852, when the yellow fever raged so furiously in New Orleans, nearly five thousand of the supporters of grog shops died before a single temperate man was attacked by the disease. In the very same year, when nine hundred died of cholera, only three were tetotallars; and when the pestilence swept off one in sixty of the entire population, of Albany, N. Y., only one in twenty-five hundred of the strictly temperate were seized with the malady. Yet, with these facts before the people, and the disease at our very doors, a very large number of our merchants and multitudes of mechanics find it impossible to leave off the use of spirituous drinks, even for a season; with so terrible malady staring them in the face, intelligent MEN WILL DRINK AND DIE!

